# I would rather be vaguely right than precisely wrong

**DOI:** 10.1080/02813432.2022.2057028

**Published:** 2022-03-30

**Authors:** Reidar Brumer Bratvold, Svein R. Kjosavik

**Affiliations:** Department of Petroleum Engineering Faculty of Science and Technology, University of Stavanger, Stavanger, Norway; The General Practice and Care Coordination Research Group, Stavanger University Hospital, Stavanger, Norway Faculty of Health Sciences, University of Stavanger, Stavanger, Norway

## How decision theory may help improve clinical practice

The only way doctors can help patients is by making decisions, whether it is to conduct tests, give advice, prescribe drugs or treatments, or refer to another doctor. Even when a GP helps the patient by listening, understanding and being compassionate, she has made a decision to do so. Such decision-making is not easy and requires an evaluation of many complex and uncertain factors. Still, if the clinician regularly makes mediocre decisions, she may never accomplish the things that are important to the patients in her care, to herself or to the healthcare system she represents. Empirical evidence demonstrates that clinicians, as well as people in general, often make suboptimal decisions [1,2]. Even when clinicians make decisions based on good quality information, they may be inconsistent and biased. Decision theory, which has been developed over more than 300 years, provides both an overall paradigm and a set of tools to help decision-makers construct and analyze models of decision situations.

## Good decisions may have poor outcomes

Judging decisions by their outcomes ignores the role of uncertainty. Actually, a majority of people confuse the quality of a decision with the quality of the outcome. If e.g. a drunk is driving home from a party, he makes a bad decision even if the trip ends well.

A clinician may practice impeccable evidence-based medicine by avoiding wasteful scans and over-diagnosis, but the consequence can be that something important will be missed. On the other hand, a clinician may order a test or a scan that results in a false-positive finding, indicating that the patient has a medical problem he does not really have. That may lead to other tests, biopsies, and even potentially harmful treatment, for a non-existing disease or an unimportant problem [3].

If these are occasional or unlikely issues, we should not worry. However, most doctors admit that they regularly order too many tests, even though they know the results won’t really help them decide how to treat their patients [4]. The medical industry spends a lot of money on data gathering and analysis to try to reduce uncertainty, but as Peter Drucker said: “*There is nothing so useless as doing efficiently that which should not be done at all.”*

Every time you let the outcome of a decision determine your assessment of that decision, slow down your thinking. *How would you judge the process leading to the decision before knowing its outcome?*

This question – How was the process? – should discipline your thinking in a way that is helpful in evaluating decisions made both by yourself and by others.

## The world is much more uncertain than you think

Every decision we make, whether in medicine or life in general, is in the face of uncertainty. The COVID-19 pandemic is a strong illustration of the world as a surprisingly uncertain place. Few considered the possibility in early 2020 that their lives would be upended for more than two years by a pandemic.

In medicine, uncertainty looms large although the people involved do not always recognize this. Atul Gawande [5] eloquently articulates this point: “As a doctor, you come to find, that the struggle for caring for people is more often with what you do not know than what you do. Medicine’s ground state is uncertain. And wisdom, for both patients and doctors, is defined by how one copes with it.”

Uncertainty without a decision is simply a worry. Quantifying uncertainty is not synonymous with decision-making, and the quality of a decision does not necessarily increase with reduced uncertainty. Uncertainty quantification or reduction creates value only to the extent that it holds the possibility of changing a decision that would otherwise be made differently. Likewise, once the decision is clear, further uncertainty reduction is a waste of resources and only serves to obfuscate the situation.

## Value can only be created through our decisions

The only purposeful way that you can influence your future is by the decisions that you make. The rest of your life happens beyond your control. When you as a GP listen with compassion to a patient, you have made a decision based on your values that this is important for the patient’s well-being. Often a patient, by talking to you, is able to move forward because you help her come to conclusions and actively engage in her decisions.

The purpose of the decision sciences is to help individuals and organizations make better decisions. Advice based on the concepts and experience in this field can be very useful to clinicians, who regularly must trade benefits and harms to choose between testing and treatment strategies. This process usually contains implicit judgments. The decision-analytic method makes the process more explicit, reproducible, and evidence-based, and has been useful in a lot of disciplines [6].

## Be clear about your values

When facing an important decision, you want to choose the best alternative. Your evaluation of the alternatives is based on your values, and these define what you hope to achieve by making the decision. However, identifying values is not easy. Research indicates that when people make a list of the values for important decisions, they typically identify less than half of their relevant values [7].

In a clinical context, it is also important to decide whose values and priorities to focus on, society’s, the patient’s, or the doctors. The core values of Nordic General Practice aim to increase our reflections on our values and priorities [8]. Without clearly articulated values, it is impossible to make good decisions.

Gawande makes this point: “Our most cruel failure in how we treat the sick and the aged is the failure to recognize that they have priorities beyond merely being safe and living longer; that the chance to shape one’s story is essential to sustaining meaning in life; that we have the opportunity to refashion our institutions, culture, and conversations in ways that transform the possibilities for the last chapters of everyone’s lives” [1].

## Improving decision-making

In life, new skills are learned and adopted. Encounters with potholes and obstacles however are inevitable, and we will periodically go astray, delaying progress. The journey to improved decision-making is no different. Along the way, complexity and uncertainty will test your determination as a decision-maker. The maxims above will help you navigate the complexity and uncertainty of important choices. Our decision-making is also challenged by the biases that each of us brings to the effort, but if we boost our awareness and take preventative actions, we can avoid the many decision traps on our journey.
